# Author Correction: Heparanase-2 protects from LPS-mediated endothelial injury by inhibiting TLR4 signalling

**DOI:** 10.1038/s41598-021-92240-w

**Published:** 2021-07-02

**Authors:** Yulia Kiyan, Sergey Tkachuk, Kestutis Kurselis, Nelli Shushakova, Klaus Stahl, Damilola Dawodu, Roman Kiyan, Boris Chichkov, Hermann Haller

**Affiliations:** 1 Department of Nephrology, Hannover Medical School, Hannover, Germany; 2 Institute of Quantum Optics, Leibniz University Hannover, Hannover, Germany; 3 Phenos GmbH, Hannover, Germany

Correction to: *Scientific Reports* 10.1038/s41598-019-50068-5, published online 19 September 2019

The Acknowledgements section in the original version of this Article was incomplete.

“We are grateful to Prof. Israel Vlodavsky (Technion, Haifa, Israel) for giving us 1c7 antibody to HPSE2. We are grateful to Prof. Fridrich Luft for critical editing the manuscript. Grants from German Federal Ministry of Education and Research (BMBF) Nr. 031A577A and 031A577B funded this research. This work was also supported by a grant for the German Research Council to H.H. Ha1388/17-1.”

now reads:

“We are grateful to Prof. Israel Vlodavsky (Technion, Haifa, Israel) for giving us 1c7 antibody to HPSE2. We are grateful to Prof. Fridrich Luft for critical editing the manuscript. Grants from German Federal Ministry of Education and Research (BMBF) Nr. 031A577A and 031A577B funded this research. This work was also supported by a grant for the German Research Council to H.H. Ha1388/17-1. This work was also supported by a grant from the Else Kröner-Fresenius-Stiftung (EKFS): Grant 2017_A96.”

The original Article has been corrected.

