# Supplementary material for: Heparanase-2 protects from LPS-mediated endothelial injury by inhibiting TLR4 signalling
Source: Sci Rep. 2019 Sep 19;9:13591. doi: 10.1038/s41598-019-50068-5 (PMC6753096; doi:10.1038/s41598-019-50068-5)
Supplement: Supplementary file 1 — supporting information [file 41598_2019_50068_MOESM1_ESM.pdf]

Supplementary information

Heparanase-2 protects from LPS-mediated endothelial injury by inhibiting TLR4 signalling

Yulia Kiyan[1], PhD; Sergey Tkachuk[1], PhD; Kestutis Kurselis[2], MS; Nelli Shushakova[3],PhD; Klaus Stahl[1], MD; Damilola Dawodu[1], PhD; Roman Kiyan[2], PhD; Boris Chichkov[2], PhD; Hermann Haller[1]*, MD*

Suppelentary Table 1. Sequences of primers used for TaqMan RT-PCR.

Name	Primer	Sequence
IL-6	Sense	5’-CTTCGGTCCAGTTGCCTTCTC-3’
	Antisense	5’-GCGGCTACATCTTTGGAATCTTC-3’
	Probe	6-FAM -CTGCTCCTGGTGTTGCCTGCTGCC- TAMRA
HPSE1	Sense	5’-ATGCTGCTGCGCTCGAAG-3’
	Antisense	5’-TCAATGGTGACGGACAGGAAC-3’
	Probe	6-FAM-CTGCCGCCGCCGCTGATGCT-TAMRA
HPSE2	Sense	5’-GGGGCTCTCTACTTGGCTCTG-3’
	Antisense	5’-GACTGTCCTGACTGGGTTCTTG-3’
	Probe	6-FAM-TGCTCCATCTCTCCCTTTCCTCCCA-TAMRA
GUSB	Sense	5’- GTGGTGCTGAGGATTGGCA-3’
	Antisense	5’-TAGCGTGTCGACCCCATTC -3’
	Probe	6-FAM- TGCCCATTCCTATGCCATCGTGTG-TAMRA

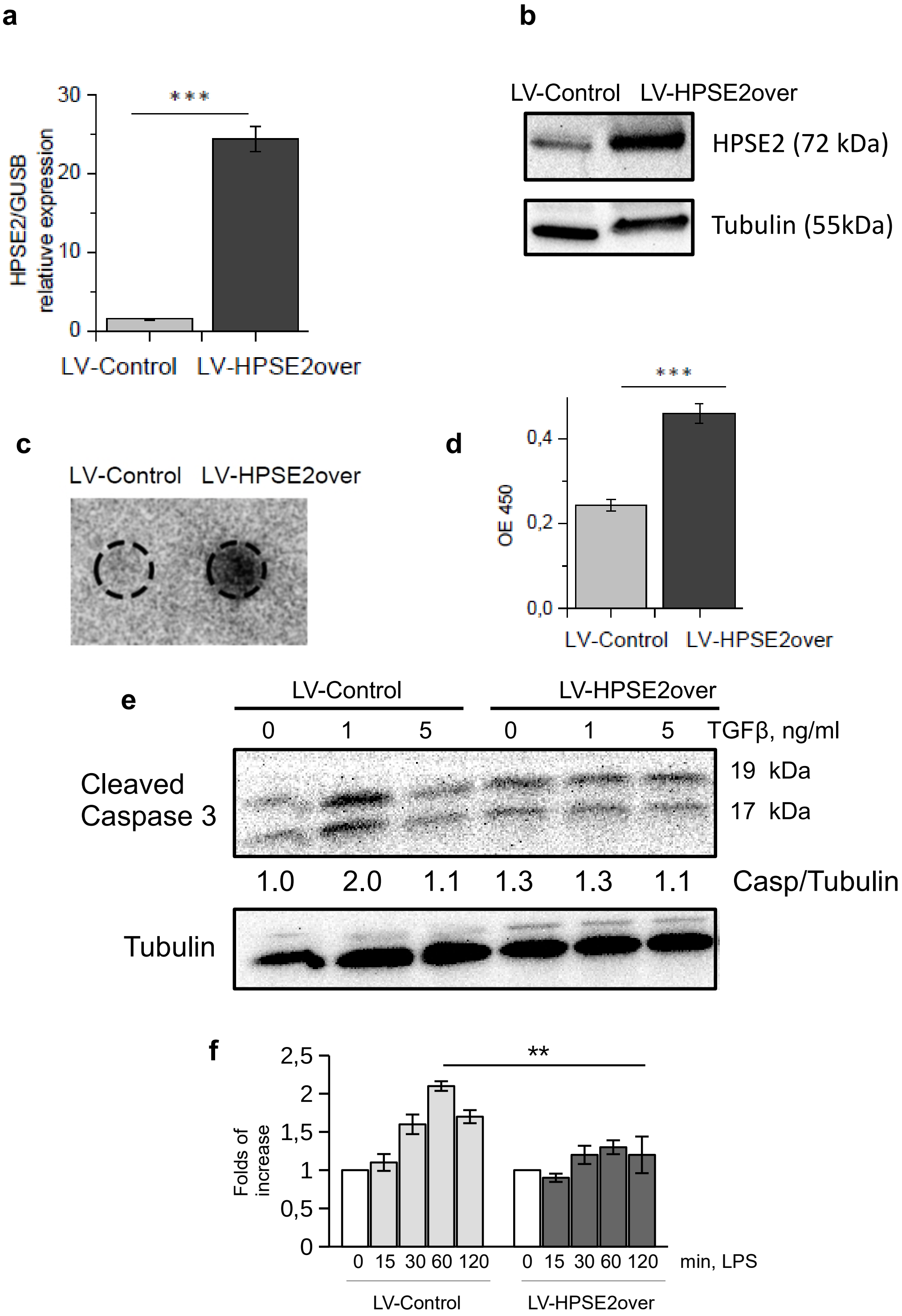

Supplementary Figure S1. a. Endothelial cells were infected with control and HPSE2-overexpression lentivirus. HPSE2 expression was analyzed by TaqMan RT-PCR. **b.** HPSE2 expression in lysates of control (LV-control) and HPSE2-overexpressing (LV-HPSE2 over) endothelial cells analysed by western blotting. **c.** HPSE2 presence in conditioned medium of control and HPSE2-over endothelial cells measured by dot blot. **d.** Proliferation of lentivirus-infected endothelial cells was determined by BrdU incorporation assay. **e.** Expression of 17 kDa and 19 kDa fragments of cleaved caspase 3 was analyzed in lentivirus-infected endothelial cells after 16h treatment with TGFβ. **f.** Quantification of western blot experiments as in Fig. 2F.

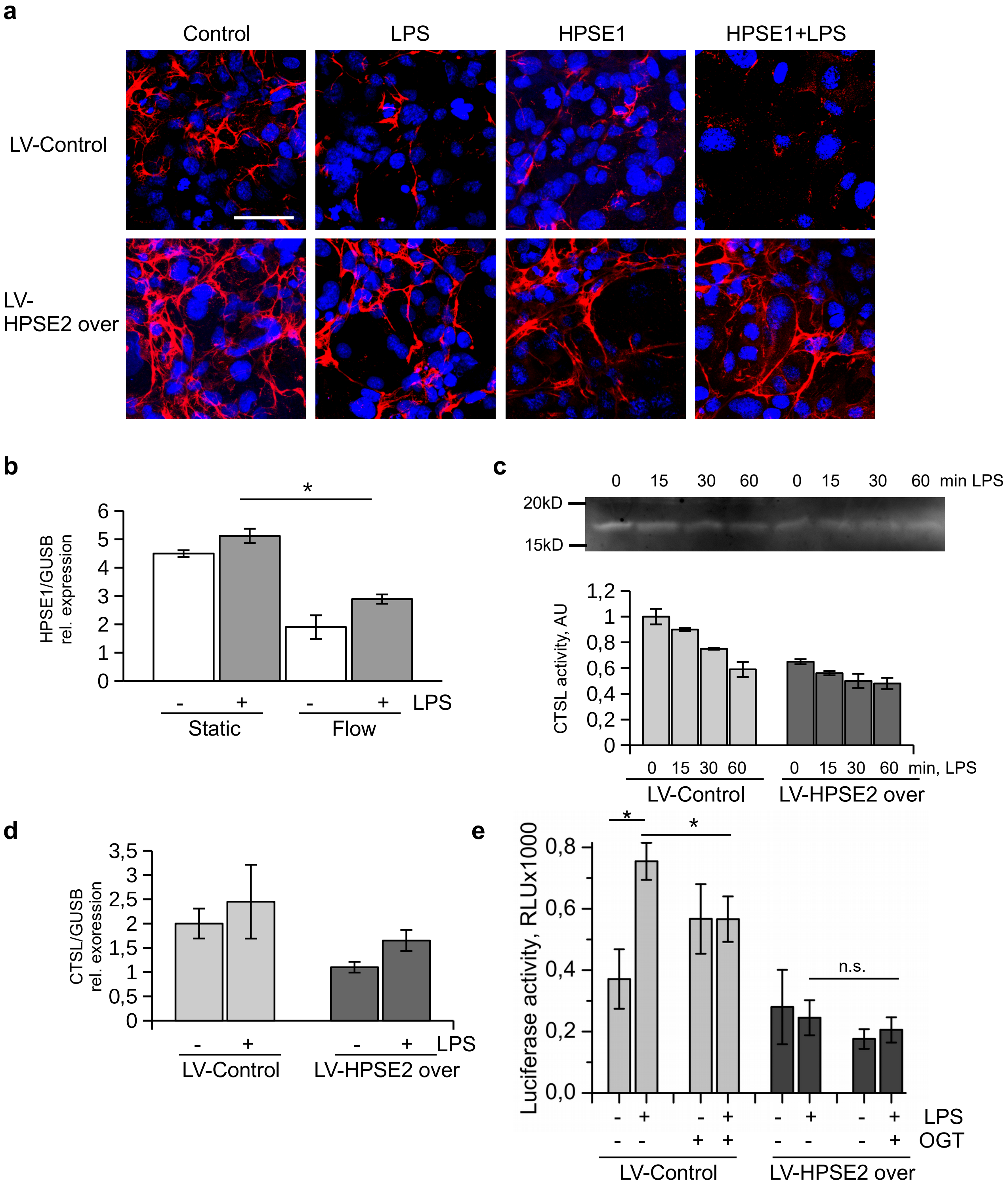

Supplementary figure S2. Protective effect of HPSE2-overexpression in endothelial cells are mediated by HPSE1 inhibition. a. Lentivirus-infected endothelial cells were cultivated under flow conditions for 3 days (38.75 dyn/cm^2), then treated with HPSE1 for 1 h, and stimulated with 100ng/ml LPS for 3 hrs. Then, cells were fixed and stained with heparan sulfate 10E4 antibody and DAPI. **b.** HPSE1 expression in endothelial cells cultivated in microlfuidic chips under static and flow conditions and stimulated with 100 ng/ml LPS for 3 hrs. **c.** Zymography in gel measurement of intracellular CTSL activity from lysates of endothelial cells. **d.** CTSL mRNA expression was analyzed by TaqMan RT-PCR. **e.** NFκB-driven expression of Gaussia luciferase in lentivirus-infected endothelial cells. Lentivirus-infected cells were pre-treated with HPSE1 inhibitor OGT 2115 and stimulated with 100ng/ml LPS for 1h.

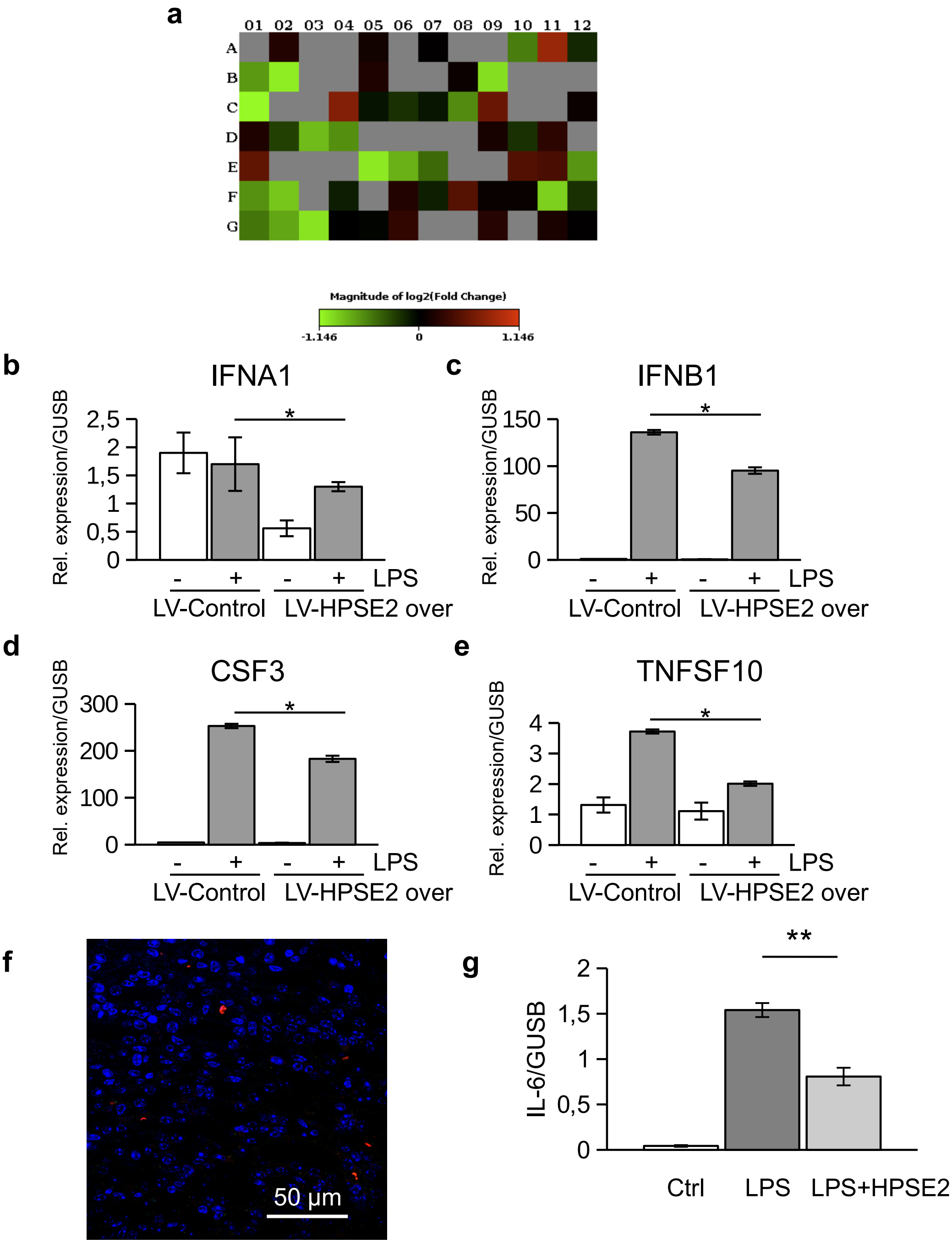

Supplementary figure S3. LPS-induced response is diminished in HPSE2-overexpressing endothelial cells. a. Heatmap of the Human Inflammation RT2 Profiler Array (Quiagen) performed from lentivirus-infected and LPS-stimulated endothelial cells. **b-e.** Expression of IFNA1, IFNB1, CSF3, TNFSF10 in the lentivirus-infected and LPS-stimulated endothelial cells was assessed by TaqMan RT-PCR. **f.** IgG negative control of mouse kidney medullary capillaries. **g.** Purified HPSE2 (50µg/ml) was added to endothelial cells in vitro before stimulation with 100ng/ml LPS for 3 hrs. IL-6 expression was assessed by TaqMan RT-PCR.

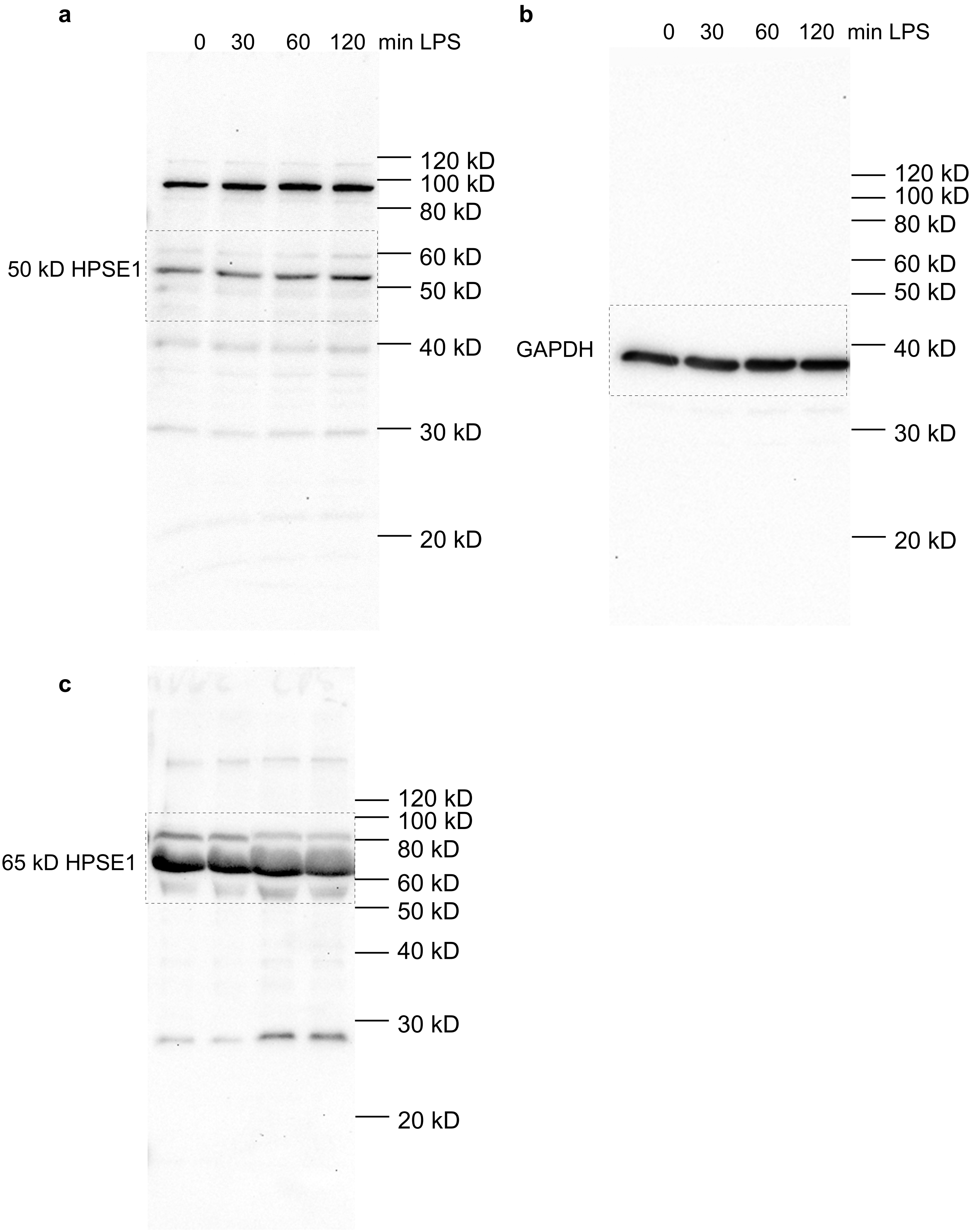

Supplementary figure S4. Full length images of the western blotting membranes used in Fig. 1 a. Fig. 1g - HPSE1. b. Fig. 1g - GAPDH. c. Fig. 1h - HPSE1.

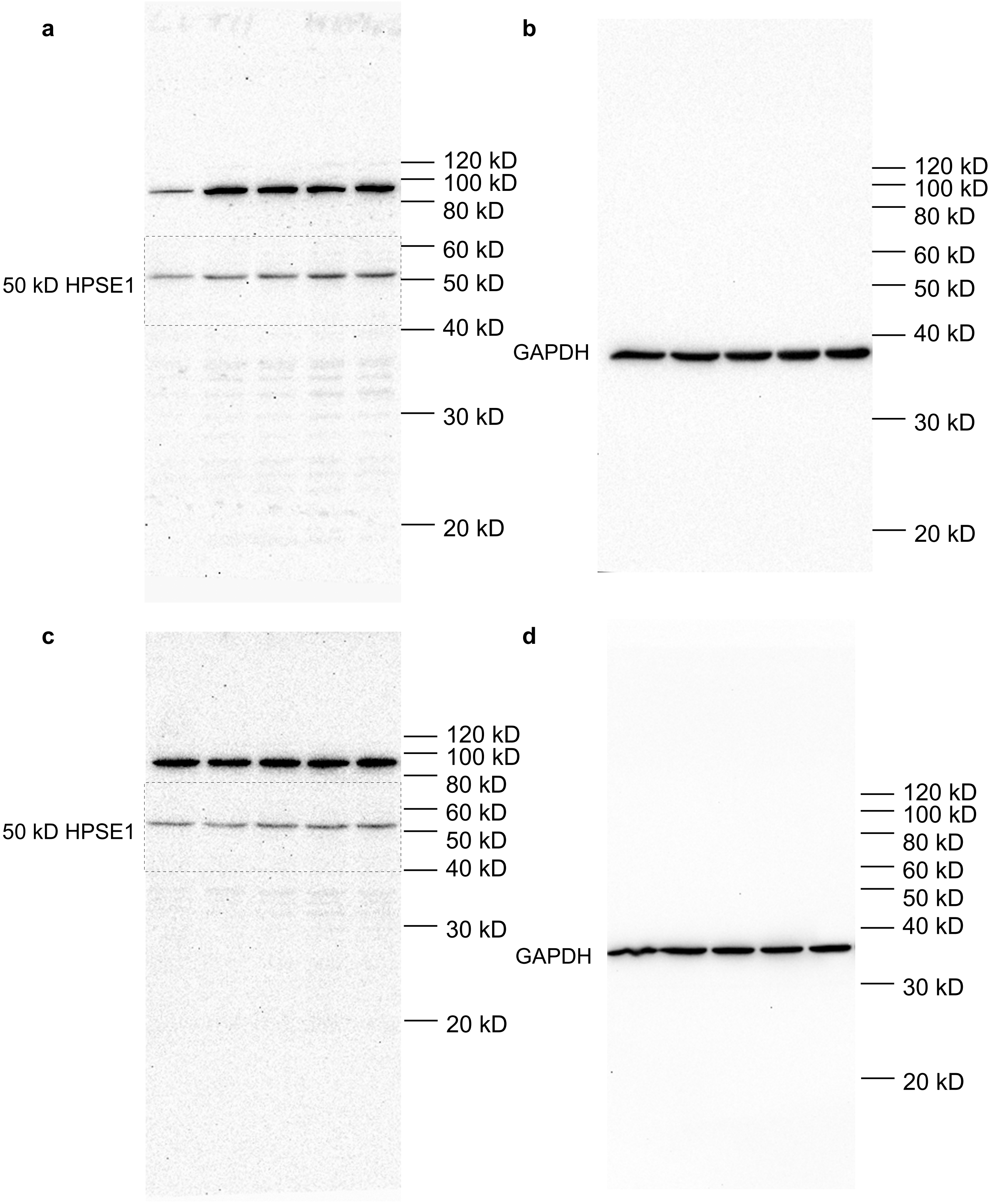

Supplementary figure S5. a. Full length images of the western blotting membranes used in Fig. 2f. a. LV-Ctrl- 50kD HPSE1. b. LV-Ctrl- GAPDH. c. LV-HPSE2-50kD HPSE2. d. LV-HPSE2-GAPDH.

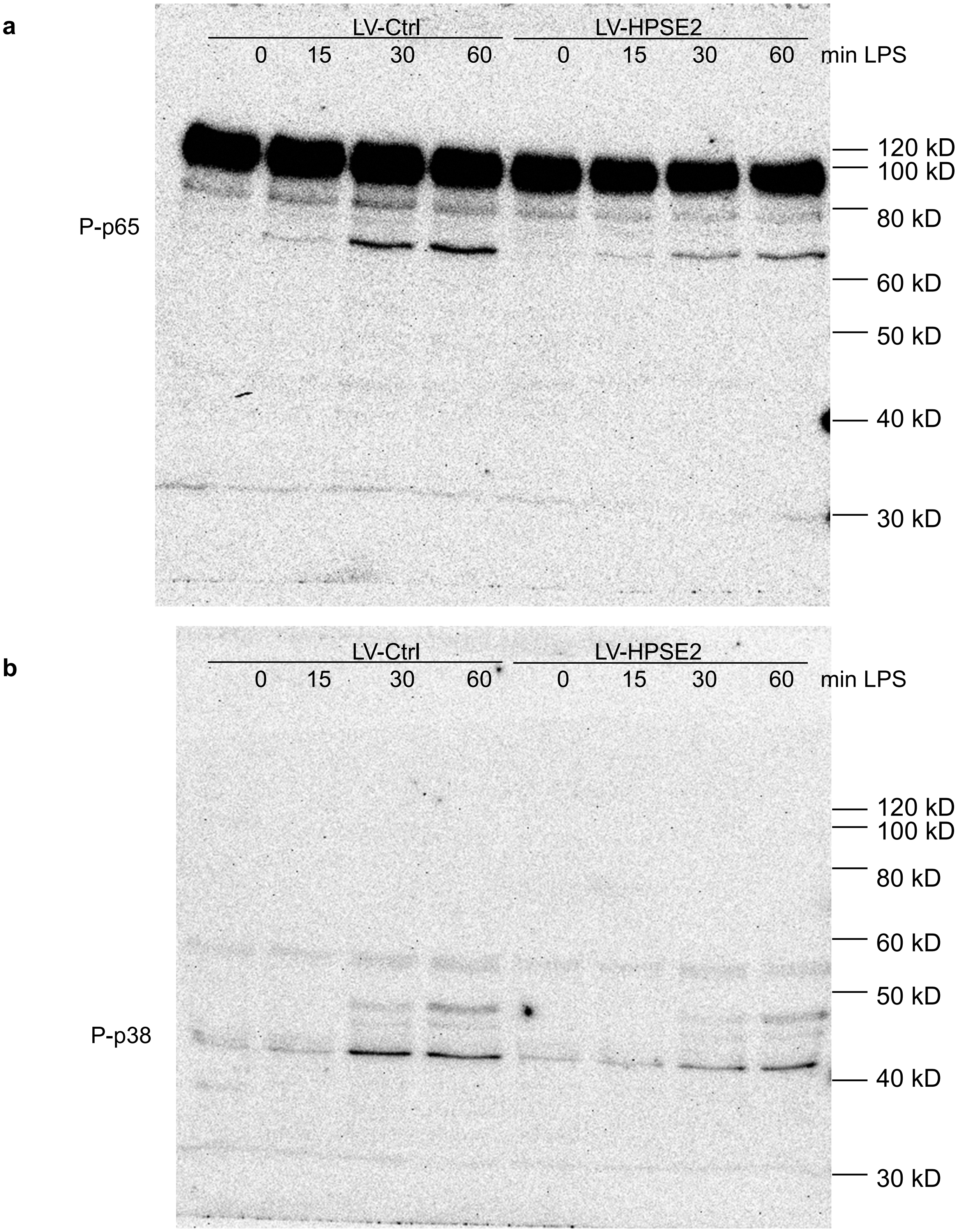

Supplementary figure S6. Full-size images of the western blotting membranes used in Fig. 3E. a. P-p65. b. P-p38.

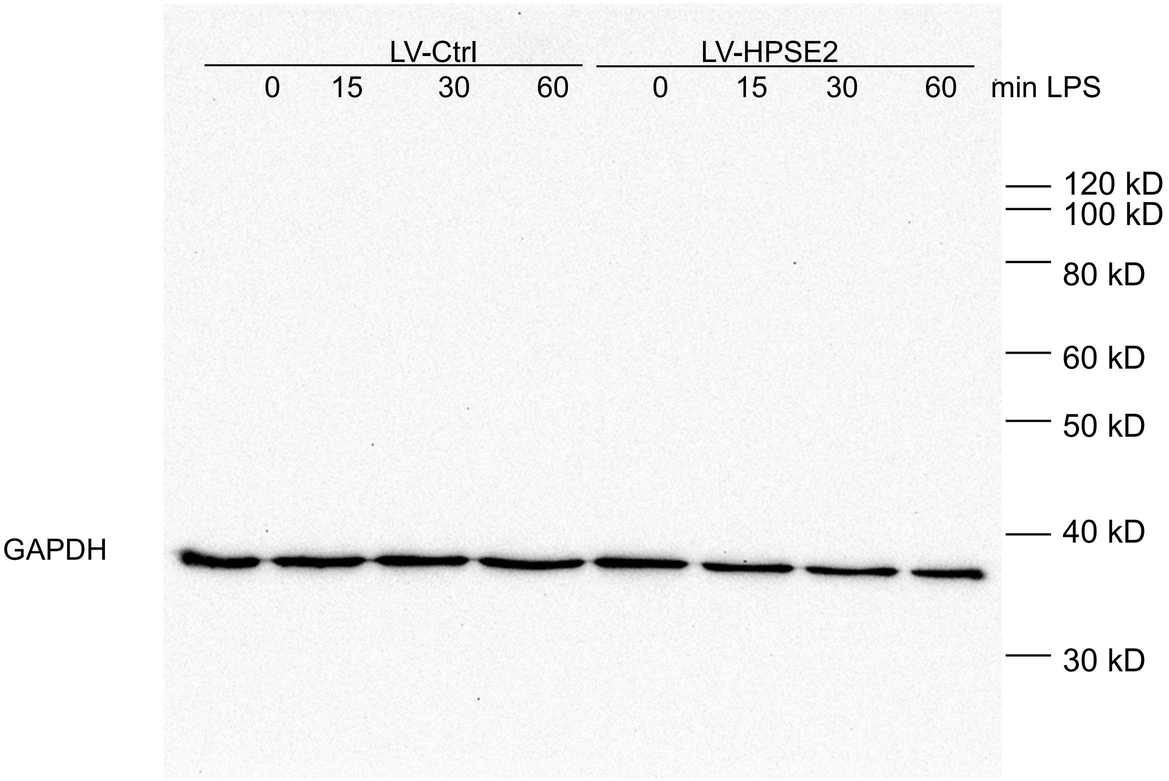

Supplementary figure S7. Full-size images of the western blotting membranes used in Fig. 3e. GAPDH.

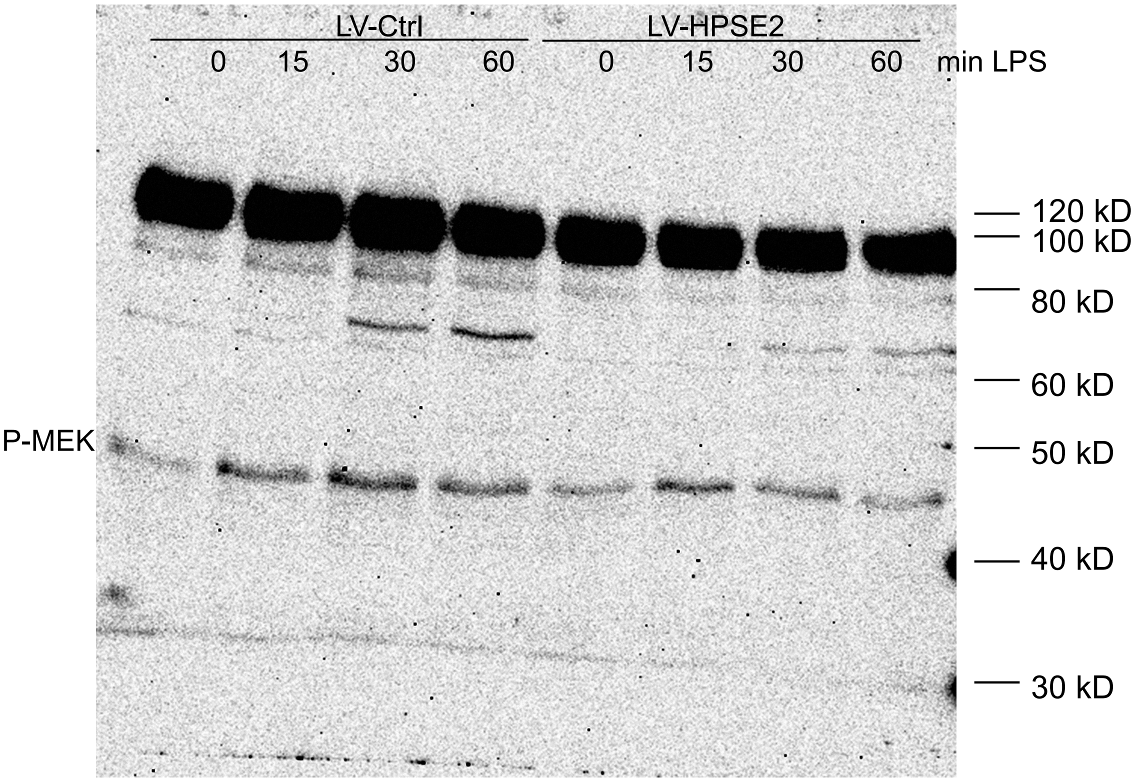

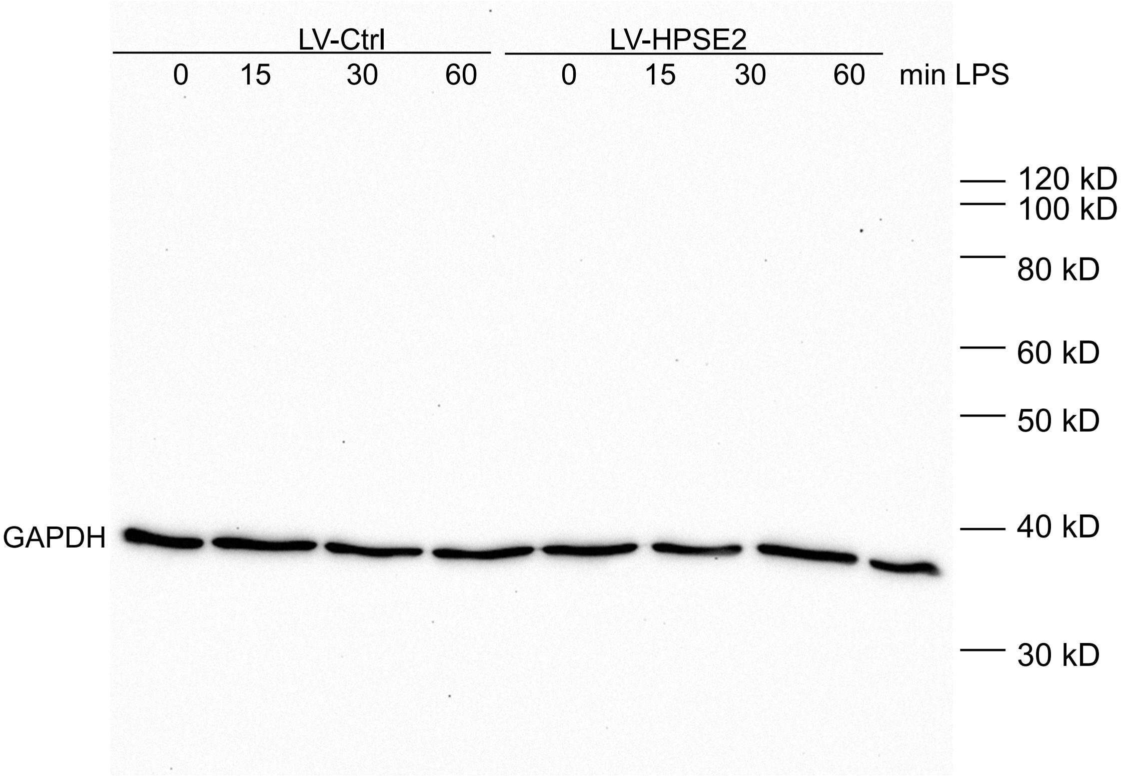

Supplementary figure S8. Full-size images of the western blotting membranes used in Fig. 3e. a. P-MEK. b. GAPDH.

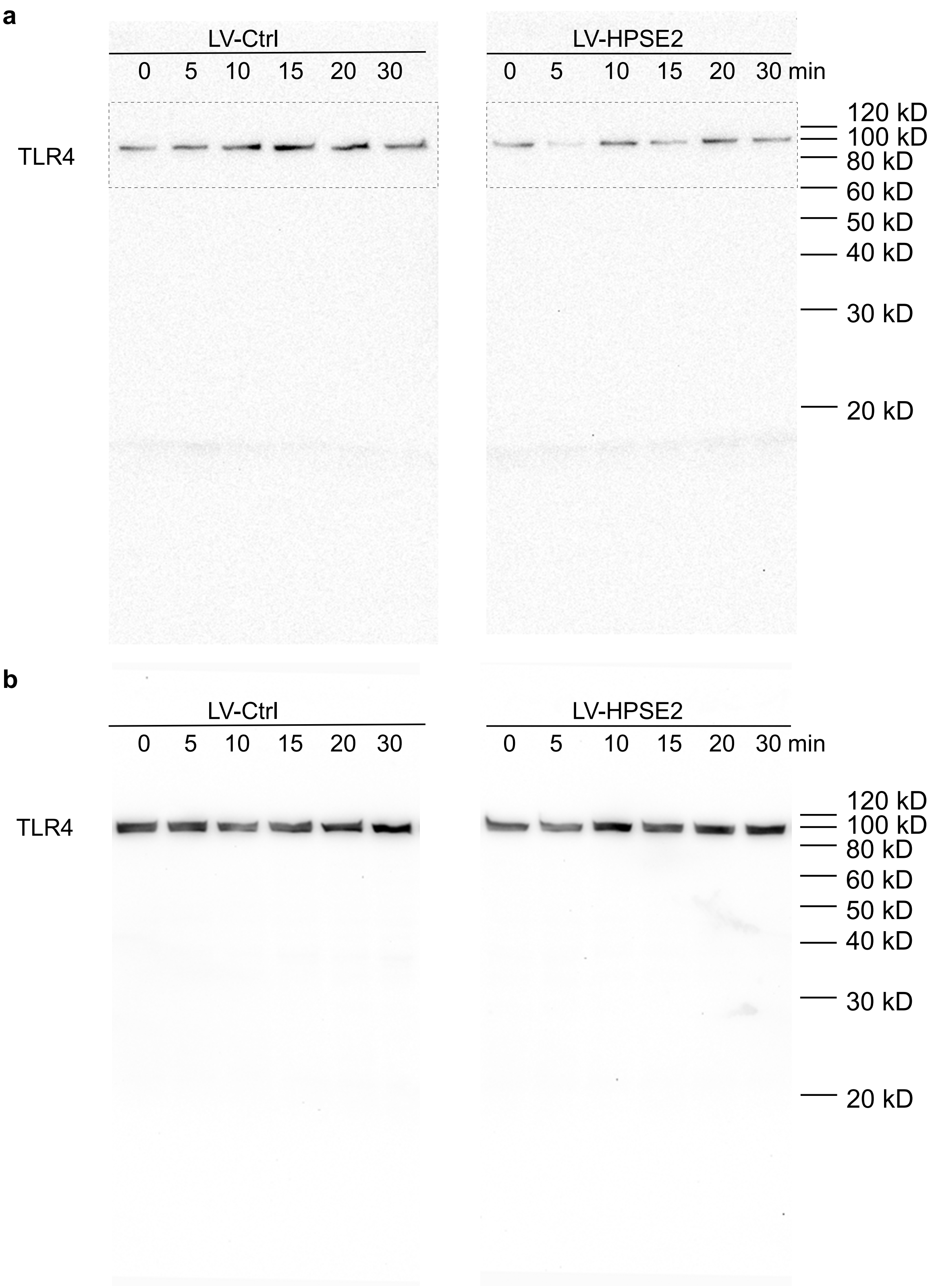

Supplementary figure S9. Full-size images of the western blotting membranes used in Fig. 6b: a. TLR4 pull-down. b. TLR4 loading.

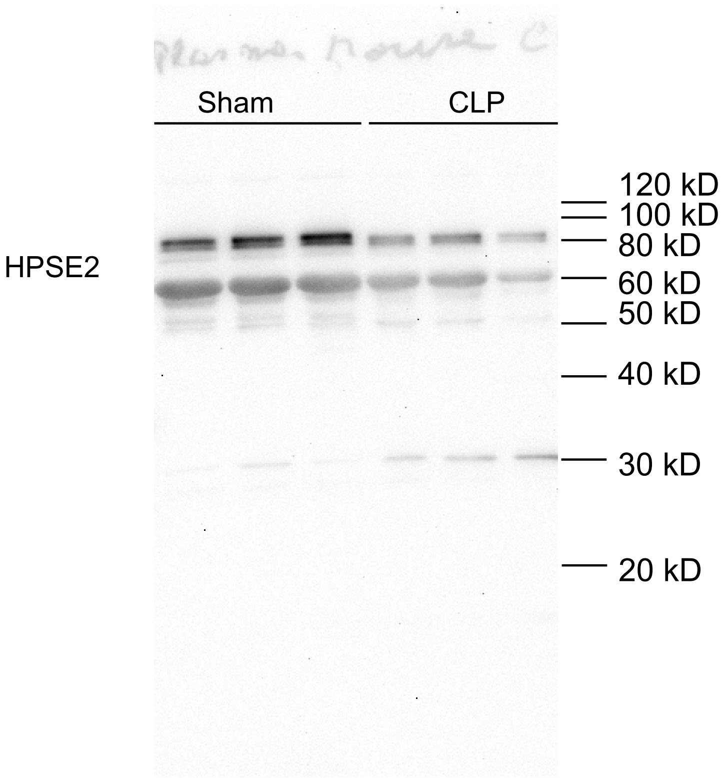

Supplementary figure S10. Full-size images of the western blotting membrane used in Fig. 7a. HPSE2.
